# Analysis of physical education based on deep learning on college students’ mental health and social adaptability

**DOI:** 10.3389/fpsyg.2022.963155

**Published:** 2022-08-11

**Authors:** Chao Wu, Ge Liu

**Affiliations:** ^1^College of Physical Education, Hubei University, Wuhan, China; ^2^Department of Sports, Zhongnan University of Economics and Law, Wuhan, China

**Keywords:** deep learning, physical education, mental health, social adaptability, physical practice education

## Abstract

With the development of learning abroad, deep learning is used in research fields. On the basis of deep learning, this article studies physical education. First, this article analyzes and explains the related concepts and current situation of physical education, and explains the measurement and definition of the mental health. Then, the function analysis algorithm of deep learning is explained and analyzed, in which the algorithm of the convolution neural network of deep learning is mainly described. Finally, through experimental analysis, it shows that the research performance of deep learning in the physical education on college students’ mental health is relatively high. At the same time, through investigation and analysis, it is proposed that physical education in deep learning can improve mental health and social adaptability relatively high. And the content of physical education should focus on increasing physical psychological education and physical practice education, which can improve college students’ mental health and social adaptability compared with other teaching contents. Therefore, when introducing deep learning, universities should strengthen the physical education of college students.

## Introduction

Deep learning is a branch of machine learning, which attempts to model high-level abstract data by using multi-layer neurons composed of complex structures or non-linear transformations. With the increase in data volume and computing power, more complex neural networks in various fields have been widely concerned and applied. This article studies in-depth learning in the neural networks, namely, popular architectural models and learning algorithms (Hao et al., 2016; Levine et al., 2016). Deep learning is introduced into the field of remote sensing geological science for data analysis. On the basis of the low-level features, the high-level output features of the network can be directly integrated into the subsequent pixel-based classification. Through the in-depth study of the actual needs of physical education and the design of the whole network input and output platform, we found the common deep learning in the physical education data analysis: traditional image processing, pixel classification and mapping, new challenges, and teaching characteristics (Zhang et al., 2016; Akhtar and Mian, 2018). Contemporary research under the background of deep learning has increasingly accepted various principles of self-determination theory. Although attention to research has increased, some assumptions of the framework have not been explored. Therefore, this study attempts to provide a more comprehensive test of physical education. This study also examines the view of modern physical education, that is, the motivation sequence contained in their framework is invariant in nature (Trudeau and Shephard, 2008; Lee et al., 2013; Gordon et al., 2016). On the model-based practice in physical education from the perspective of social adaptation. In the connection between the critical methods of physical education, we think that depth models are useful tools worthy of integration into physical education, but we are worried that they should redefine the purpose of physical education. At last, it shows that the deep learning model can be a useful tool for thinking about physical education, but the practice based on the deep learning model cannot replace the thoughtful and thorough physical education curriculum (Bekiari and Tsaggopoulou, 2016; Landi et al., 2016; Olson et al., 2016). On the mental health and psychological well-being of physical education, personnel shows that. This review evaluates the basis of the mental health and social adaptability of elite athletes. The results show that compared with the general population, people who have experienced physical education have a lower risk of mental disorders (i.e., anxiety and depression). It shows that physical education has a positive role in promoting mental health (Mcardle et al., 2007; Carson et al., 2016; Fisher et al., 2016; Rice et al., 2016; Chen et al., 2021).

## Introduction of deep learning algorithm

### Deep learning model function algorithm

The multivariate logistic regression is widely used in non-linear classification in the fields of deep learning and machine learning. Improved linear regression model. The main advantage of this model is that it has strong explanatory power compared with other classification algorithms. Combined with the excellent classification ability and prediction ability of deep learning, multivariate logistic regression algorithm is one of the most popular deep learning algorithms in the industry, which is widely used in pattern recognition, pattern prediction, and text classification. The principle is that the dimension of one particle vector is the same as that of another biological vector, and the modified sample set is [0, 1]. The probability distributions of all types of overlapping soft maximum function and the probability distribution of the whole area are given, where the function expression is:


(1) $$S\,o\,f\,t\,\max\,\left(x_{j}\right)=\frac{e^{x\,j}}{\sum_{k-1}^{k}e^{x_{k}}}$$

The result predicted by the Softmax function is the probability that a sample belongs to the corresponding kind. The formula is as follows:


(2) $$h_{o}\,\left(x\right)\,\frac{1}{\sum_{i-1}^{k}e^{\theta_{i}^{T}\,x}}\,\left[\begin{array}{c} e^{\theta_{i}^{T}\,x}\\ \ldots \\ e^{\theta_{i}^{T}\,x} \end{array}\right]$$

The output set of the Softmax function is a *K*-dimensional probability vector, and all the probabilities add up to 100%. Therefore, the multivariate logistic regression model predicts the probability that the existing data belongs to various types, and predicts the type with the highest probability as the type of the data, so as to realize the classification of sample sets. In the process of multivariate logistic regression model classifying circuit states, the training sets in the sample set are trained in the classification model, and the probability distributions of different states are obtained through mapping relations.

The neighborhood *K* algorithm is used for classification and data mining. The KNN algorithm is an effective data classification model, and its function is to complete data classification and prediction, namely, the dependent prediction of discrete variables and continuous variables. The KNN algorithm is insensitive to the size and diversity of samples and does not depend on the distribution of samples, which makes it a very intuitive machine learning model. The principle of the KNN algorithm is the sample set. The most recent particle data in the sample data have the same characteristics, so the sample data have the same characteristics and are considered to be the same species. The algorithm determines the region of prediction points according to the known parameters of the new prediction region. In practical application, the type of test sample is usually determined by calculating the distance between the sample and the known sample and taking the known sample area closest to the known sample as the judgment basis. The expressions of the Euclidean distance and Manhattan distance of its leading algorithm are as follows:


(3) $$d_{A,B}=\sqrt{{\left(x_{1}-x_{2}\right)}^{2}+{\left(y_{1}-y_{2}\right)}^{2}}$$

(4) $$d_{A,B}=\left|x_{1}-x_{2}\right|+\left|y_{1}-y_{2}\right|$$

The K-proximity algorithm uses the aforementioned two distances to measure the similarity between samples. The data processing process of the algorithm is to calculate the distance between the test eigenvalue and each training eigenvalue, sort according to the increasing distance between points, select the smallest K points in the length sequence, count the distribution number of the states belonging to the first K points, and then judge the state of the input eigenvalue as the type with the most states. Its essence is to calculate and compare the distance between the measured and known eigenvalues. The formula for calculating the predicted type of test eigenvalue is:


(5) $$\bar{y}=\left(\sum_{i=1}^{k}y\right)/k$$

(6) $$M\,S\,E=\frac{1}{N}\,\sum_{i=1}^{N}{\left(Y^{\left(i\right)}-f\,\left(x^{\left(i\right)}\right)\right)}^{2}$$

The activation function is a kind of function that mostly exists in the hidden layer. The activation function is used mainly to add non-linear factors of the model, and at the same time, it can reduce the parameters of the model, so as to speed up the convergence and improve its learning ability. If there is no activation function, then each layer of the neural network is equal to multiplying the matrix, and the multi-layer neural network only multiplies the matrix continuously. There are many kinds of commonly used activation functions. The following is an introduction to the commonly used activation functions: Sigmoid is a non-linear activation function, which has a domain of R and a range of (0, 1). In general, sigmoid is used when dealing with the binary classification problems and regression problems, and it will have a better effect when added to the output layer. Where the function expression is:


(7) $$S\,\left(x\right)=\frac{1}{1+e^{-x}}+\frac{e^{y}+e^{x}\,\left(1-x\right)}{x}$$

At this point, the effect of the model is equal to a classifier, which shows the probability generated by each category. The expression for this activation function is:


(8) $$S_{i}=\frac{e^{v_{i}}}{\sum_{j}^{c}e^{v_{j}}}$$

Relu is a unilateral inhibition activation function, which is mostly used to increase the non-linear relationship of each layer of the network. Its function image is very simple, and the expression of this function is as follows:


(9) R⁢(x)=max⁡(0,x)

The advantage of this activation function is that when the independent variable is a non-positive real number, its function value is always 0 in Formula 9, and when the independent variable is a positive real number, its function value increases with the increase of the variable. Tanh is a hyperbolic tangent activation function, which looks a little like the Sigmoid function, so they all have saturation region, which will cause gradient dispersion problems. The difference is that the range changes from (0, 1) to (−1, 1), and Tanh can be regarded as the transformation of Sigmoid. The expression of this activation function is:


(10) $$\tanh\left(x\right)\,\frac{2}{1+e^{-2\,x}}-1$$

### Deep learning network model

The complex neural network of snake venom learning processes information by adjusting the interaction between nodes in the network. In a biological neural network, each neuron is connected with other neurons, and when a neuron receives a signal, it makes corresponding feedback. However, the loss function is for a single sample, and the operation for a single sample cannot guarantee universality or generalization. Therefore, we use the cost function for multiple samples, the average value of the loss function for samples. However, the loss function is for a single sample, and the operation for a single sample cannot guarantee universality or generalization. Its expression formula is:


(11) $$J\,\left(W,b\right)=\frac{1}{m}\,\sum_{i=1}^{m}L\,\left(y^{-\left(i\right)},y^{\left(i\right)}\right)$$

*W* and *B* are unknown parameters, which need to be trained and optimized repeatedly. The cost function is a function of the coefficients *W* and *B*. The goal of our study is to calculate the best values of *W* and *B* many times, so that the cost function approaches zero as much as possible. To this end, we use the gradient descent algorithm to move a small amount of the gradient descent in the opposite direction, and the iterative update expression is:


(12) $$W_{i+1}=W_{t}-a\,\frac{\partial_{J}\left(W_{t},b\right)}{\partial W_{t}}$$

(13) $$b_{i+1}=b_{t}-a\,\frac{\partial_{J}\left(W,b_{t}\right)}{\partial b_{t}}$$

The training methods of the neural network include forward propagation method and backward propagation method. Predict the output process through the neural network, from input and output results to forward propagation; Parameters *W* and *B* are iteratively updated from output to input. A classical neural network consists of the input layer, output layer, and hidden layer. We might as well assume an R-layer neural network. Then, the output of this neural network can be calculated layer by layer by the following formula:


(14) $$h^{k+1}=\sigma^{k}\,\left(Z^{k}\right)=\sigma^{k}\,\left(W^{k}\,h^{k}+b^{k}\right)$$

The weight and offset can be calculated in the forward propagation process of deep learning. According to the weight and offset, it can be sorted out as follows:


(15) $$\left\{ \begin{array}{c} \frac{\partial h^{k+1}}{\partial h^{k}}={\left(\sigma^{k}\right)}^{\prime }\,h^{k}\\ \frac{\partial h^{k+1}}{\partial W^{k}}={\left(\sigma^{k}\right)}^{\prime }\,h^{k} \end{array}\right.$$

Deep learning convolution neural network convolution integral is internal convolution and external convolution. We use the forward propagation of the internal convolution computer network and the back propagation of the external convolution computer network. The concrete process of convolution is as follows: the input characteristic graph is Matrix A, the convolution kernel is Matrix B, and the convolution kernel moves in a certain step. Every time it moves, it is convolved with the overlapping part in the feature map A, and then a value is obtained. After the convolution kernel moves, all the values are combined into inner convolution C in sequence, that is, the green matrix in the map, in which the size of the convolution kernel, the number of channels, the step size, and whether the feature map needs to be filled are all the super parameters that need to be set manually. According to the convolution chain rule, there are:


(16) $$\frac{\partial E}{\partial A}=\frac{\partial E}{\partial C}\cdot \frac{\partial C}{\partial A}=A*\frac{\partial E}{\partial C}$$

(17) $$\frac{\partial E}{\partial A}=\frac{\partial E}{\partial C}\cdot \frac{\partial C}{\partial A}=\frac{\partial E}{\partial C}*r\,o\,t\,180\,\left(B\right)$$

There are different units and shapes among different blocks of deep learning standard convolution nerve, such as different convolution kernels (convolution kernel size, channel number, step size, etc.), different activation functions, different pooling operations, or non-existence. The recursive expression of the network structure is:


(18) $$\frac{\partial J\,\left(W,b\right)}{\partial Z^{k}}=\frac{\partial J\,\left(W,b\right)}{\partial Z^{k+1}}\,W^{k+1}\,{\left(\sigma^{k}\right)}^{\prime }$$

Convolution propagation algorithm is a neural network method based on error minimization, which regresses to propagation and control matrix based on gradient descent method. Calculate the gradient of the hidden layer and calculate the remaining calculation amount of each neuron in all the layers. The specific formula is:


(19) $$\delta^{l}=\left({\left(w^{l+1}\right)}^{T}*\delta^{l+1}\right)\cdot f^{\prime }\,\left(x^{l}\right)$$

The residual error of the output layer is determined by this layer and can be expressed as:


(20) $$\delta^{L}=f^{\prime }\,\left(x^{L}\right)\cdot \left(y^{\prime \prime }-t^{\prime \prime }\right)$$

Deep learning cyclic neural network can process sequence data well. Obviously, the length of sequence signals will change with different scenes, while the input size of traditional neural networks is fixed, which cannot handle this situation. In addition, it is difficult for traditional models to deal with long-distance dependencies in sequence signals. Compared with the traditional network, the structure of RNN has changed greatly. The traditional network is connected layer by layer, but the neurons between layers are not connected. Its expression form is:


(21) $$\sum_{j=1}^{s}KL\left(\text{ρ}||\bar{{\text{ρ}}_{j}}\right)=\left(\text{ρ}\log=\frac{\text{ρ}}{\bar{\text{ρ}}}+1\left(1-\text{ρ}\right)\log\frac{1-\text{ρ}}{1-\bar{{\text{ρ}}_{j}}}\right)$$

The average activation value of hidden layer nodes meets:


(22) $$\bar{{\text{ρ}}_{j}}=\frac{1}{m}\,\sum_{i=1}^{m}d_{i\,j}$$

## Analysis of physical education on college students’ mental health and social adaptability under deep learning

### Influence of physical education on mental health under deep learning

**FIGURE 1:**
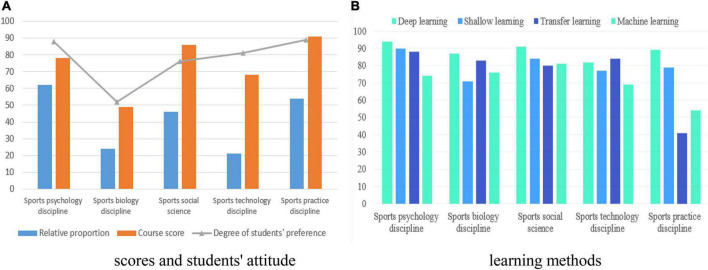
Deep learning on the physical education content. **(A)** Scores and students’ attitude; **(B)** learning methods.

Although it takes longer than machine learning and transfer learning, deep learning is more descriptive in studying the mental health and social adaptability of college students in physical education, because a large amount of data need to be collected in the research, so that it is descriptive and not random. Therefore, we apply deep learning to all the aspects of physical education, and we will study the content of physical education through deep learning and other learning methods. The results show that deep learning is more sufficient in studying educational psychology, sports biology, sports society, and sports technology data. Among them, the content of physical education in physical education based on deep learning is shown in Figure 1A. The research on physical education content by its deep learning and other learning methods is shown in Figure 1B:

First, we use the model to study the mental health of college students, which is that the evaluation index of college students’ mental health level is strong, many factors, high sensitivity, anxiety, depression, psychological imbalance, and poor-psychological state of college students’ psychological instability. The Five-grade scoring method was used. The lower the score, the better the mental health. The system has good reliability and effectiveness. At first, we randomly select college students from various majors in major universities and set up two groups, namely, the experimental group and the control group. Among them, both the experimental group and the control group have not experienced systematic physical education and paid no attention to it.

In Table 1, it can be found that the mental health degree of the experimental group and the control group is not correct before adopting systematic physical education, which indicates that the two groups can be used for the relative experiment. Then, these students learned a series of basic theories from psychology courses, sports biology research, sports social sciences, and sports technology, and mastered the basic theories and skills of sports in-depth. Through systematic physical education, the experimental group and the control group carried out psychological tests.

**TABLE 1:** Mental health analysis of experimental group and control group before and after the experiment.

Indicators	Group	Number	Mean		S.D.		T		P	
			After	Before	After	Before	After	Before	After	Before
Obsessive factor	Experimental group	42	11.1	11.3	2.5	2.7	−0.111	−0.792	0.431	0.847
	Control group	43	11.5	11.4	3.6	3.4				
Paranoid factor	Experimental group	42	10.6	11.2	2.9	3.1	−0.287	−0.282	0.779	0.775
	Control group	43	10.8	11.9	4.7	5.3				
Hostile factor	Experimental group	42	9.4	11.2	3.3	3.6	0.063	−2.194	0.321	0.549
	Control group	43	11.6	11.7	4.3	6.3				
Interpersonal relationship	Experimental group	42	9.8	12.4	3.1	3.6	−0.052	−2.175	0.332	0.907
	Control group	43	11.9	12.5	4.3	5.2				
Depression factor	Experimental group	42	9.8	10.7	3.2	3.9	−0.117	−1.096	0.276	0.907
	Control group	43	10.7	10.8	4.4	3.7				
Anxiety factor	Experimental group	42	8.9	9.6	4	4.5	0.325	−0.556	0.579	0.746
	Control group	43	9.4	9.3	3.2	3.1				
Learning pressure	Experimental group	42	11.9	14.7	3.5	5.9	0.264	−2.313	0.231	0.793
	Control group	43	14.6	14.4	7.6	4.8				
Maladjustment	Experimental group	42	9.1	9.9	3	3.9	−1.131	−1.262	0.215	0.262
	Control group	43	10	10.7	2.7	2.7				
Emotional instability	Experimental group	42	10.1	12.7	2.7	3.8	1.14	−2.224	0.297	0.26
	Control group	43	12.2	12.7	5.5	5.1				
Psychological imbalance	Experimental group	42	8.7	9.5	2.8	2.9	0.009	−1.587	0.117	0.993
	Control group	43	9.6	9.7	2.3	3.8				

By comparing the mental health analysis of the experimental group and the control group before and after systematic physical education, we can get four dimensions from the aforementioned data, that is, physical education teaching mode can improve students’ output, interpersonal communication, frustration education, and conventional thinking. Obsessive compulsion, paranoia, depression, and anxiety are four aspects of hostility, and social, stress, and emotional stability have great influence. The three dimensions of maladjustment, emotional instability, and psychological imbalance are relatively less obvious. In general, physical education plays a very important role in optimizing the mental health of college students. Physical education is beneficial to the healthy development of college students’ psychology.

### Analysis of physical education’s adaptability to society under deep learning

**FIGURE 2:**
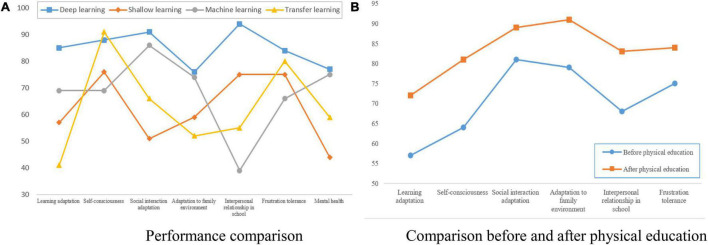
Deep learning in social adaptability analysis. **(A)** Performance comparison; **(B)** comparison before and after physical education.

With the development of deep learning, deep learning has been applied to various research fields. At the same time, deep learning has quite optimistic research significance for massive data analysis and processing. Therefore, when we study the analysis of physical education on social adaptability, we will introduce deep learning, because when we study the analysis of physical education on social adaptability, we will investigate a large amount of data, and we need deep learning to analyze these massive data. When analyzing these college students’ social adaptability, we compare the adaptability performance of college students under physical education through deep learning and shallow learning. The experimental results show that the research data of deep learning on college students’ social adaptability in physical education is more reliable than transfer learning, shallow learning, and machine learning, which is generally more conducive to the research on college students’ adaptability performance in physical education. The comparison results are shown in Figure 2:

From Figure 2, there is a correlation between social adaptability and psychological defense mechanism, and improving psychological defense mechanisms can better adapt to society.

The research shows that the data research and experimental analysis of deep learning on social adaptability are generally higher than other learning methods. College students’ social adaptation includes six dimensions: self-regulation, learning adaptation, social adaptation, pressure bearing, school adaptation, family adaptation, and environment adaptation. In these six dimensions, the total score is 100 points, and the higher the score, the stronger is its adaptability. Therefore, in order to investigate the analysis of social adaptability of physical education based on deep learning, we randomly selected college students for investigation. And statistics of college students’ social adaptability before and after physical education. At last, deep learning is also used to analyze and predict the data. Through in-depth study of physical education research, students’ mental health and social adaptability can be improved, the hostility and pressure of education can be reduced, mental health and self-awareness can be improved, and social adaptability and family ability can be improved. Physical education is not suitable for all the sports because of a single adventure. Sports and health education should focus on selecting professional and sports teams according to the local conditions. In the implementation of the physical education teaching mode, we should pay attention to the core content of the teaching evaluation process, and evaluate the teaching process through tools to ensure its effectiveness.

### Analysis of physical education content on mental health and social adaptability

**FIGURE 3:**
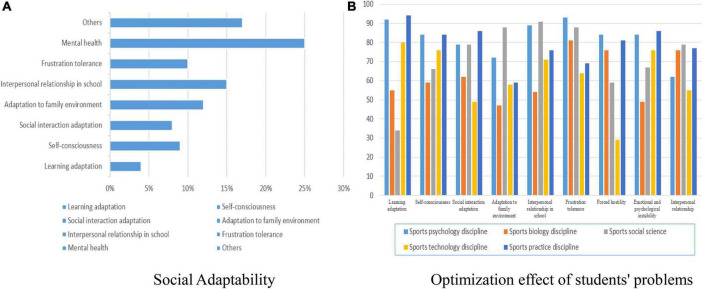
Optimization effect and social adaptability. **(A)** Social adaptability; **(B)** optimization effect of students’ problems.

Physical education has a good overall evaluation of mental health and social adaptability, However, sports psychological education and sports practical education have a better evaluation of mental health. Sports social education and sports practice education have a better evaluation of social communication adaptability, and sports psychological education, sports social education, and sports practice education have a better evaluation of intramural interpersonal relationships in social adaptability. In terms of family environment adaptability in social adaptability, sports social education and sports practice education have better evaluation. Therefore, in physical education in universities, it is necessary to increase the content of physical psychological education and physical practice education for college students. So, we continue to follow-up the investigation. It is found that students have problems in some aspects. In general, most contemporary college students have problems with mental health and hard ability of social adaptation. But, the problem is not very big. To treat psychology through sports, the first is the science of physical education, and the second is the social science of sports. The survey shows that sports psychology and sports practice are good for improving college students’ mental health ability and social practice ability. Sports biology and sports technology are relatively poor in the promotion. The research and investigation show that we should prescribe the right medicine in carrying out physical education and perform more physical psychology and physical practice disciplines to improve the mental health and social adaptability of college students. Studies have shown that students have mental health problems and social adaptation problems, as shown in Figure 3A. The promotion ability of each content of physical education relative to each factor of mental health and social adaptability is shown in Figure 3B.

Physical education can improve mental health and social adaptability compared with music education and art education, but the refreshing effect of music education and art education is not bad, so colleges and universities should diversify their education for the college students. Students with low-mental health level should actively participate in physical education, so as to adjust their state.

## Concluding

With the deep learning, deep learning has basically replaced the previous technology and made great breakthroughs in pattern recognition, speech recognition, and information retrieval. In-depth research has been paid more and more attention by the people. At the same time, in order to improve the learning mechanism, a more classical deep learning framework is proposed. Therefore, in-depth research will be introduced into the analysis of students’ mental health and social adaptation in physical education. In recent years, various foreign standards have been introduced into spina bifida curriculum, which provides many ideas for the effective implementation of physical education and health curriculum. Physical education mode is a teaching mode with the main goal of strengthening the social adaptability of college students’ mental health. It focuses on establishing the contents of physical practice education and physical psychology education, increasing the extracurricular activities of college students, and strengthening through physical education. Therefore, the physical education of deep learning is beneficial to the improvement of college students’ mental health and social adaptability.

## Data availability statement

The original contributions presented in this study are included in the article/supplementary material, further inquiries can be directed to the corresponding author.

## Author contributions

Both authors participated in the preparation and presentation of the manuscript.
